# Three-Dimensional Supermacroporous Carrageenan-Gelatin Cryogel Matrix for Tissue Engineering Applications

**DOI:** 10.1155/2013/478279

**Published:** 2013-07-07

**Authors:** Archana Sharma, Sumrita Bhat, Tanushree Vishnoi, Vijayashree Nayak, Ashok Kumar

**Affiliations:** ^1^Department of Biological Sciences, Birla Institute of Technology and Science, Pilani, K.K Birla Goa Campus, Goa 403726, India; ^2^Department of Biological Sciences and Bioengineering, Indian Institute of Technology Kanpur, Kanpur, Uttar Pradesh 208016, India

## Abstract

A tissue-engineered polymeric scaffold should provide suitable macroporous structure similar to that of extracellular matrix which can induce cellular activities and guide tissue regeneration. Cryogelation is a technique in which appropriate monomers or polymeric precursors frozen at sub-zero temperature leads to the formation of supermacroporous cryogel matrices. In this study carrageenan-gelatin (natural polymers) cryogels were synthesized by using glutaraldehyde and 1-ethyl-3-[3-dimethylaminopropyl] carbodiimide hydrochloride and *N*-hydroxysuccinimide (EDC-NHS) as crosslinking agent at optimum concentrations. Matrices showed large and interconnected pores which were in the range of 60–100 **μ**m diameter. Unconfined compression analysis showed elasticity and physical integrity of all cryogels, as these matrices regained their original length after 90% compressing from the original size. Moreover Young's modulus was found to be in the range of 4–11 kPa for the dry cryogel sections. These cryogels also exhibited good *in vitro* degradation capacity at 37 °C within 4 weeks of incubation. Supermacroporous carrageenan-gelatin cryogels showed efficient cell adherence and proliferation of Cos-7 cells which was examined by SEM. PI nuclear stain was used to observe cell-matrix interaction. Cytotoxicity of the scaffolds was checked by MTT assay which showed that cryogels are biocompatible and act as a potential material for tissue engineering and regenerative medicine.

## 1. Introduction

Tissue engineering has emerged as an alternative approach for the repair or regeneration of damaged tissues or organs. In the area of tissue engineering, one of the major research themes is fabrication of scaffold which provides an adequate three-dimensional (3D) support for transplanted cells. Cultivation of the cells on 3D matrices provides certain advantages like increase of the cell survival, guide cell differentiation, and cell fate *in vitro*. Scaffold is the most important component of tissue engineering; therefore various fabrication techniques are being explored for the fabrication of porous 3D scaffolds using natural or synthetic polymers. Conventional scaffolding technologies include gas foaming, fibre meshes, phase separation, melt moulding, solvent casting, particulate leaching, and emulsion freeze drying [1]. Among the conventional techniques, freeze drying is widely being used for fabricating scaffolds for tissue engineering applications [2]. But due to some limitations with these techniques such as incapability of controlling pore size, uneven pore distribution, and poor mechanical integrity, recently new scaffold designing approach has been developed known as “*cryogelation*.*”* This technique synthesizes scaffolds as supermacroporous matrices which have shown applicability in the area of tissue engineering and regenerative medicine [3].

“Cryogels” are supermacroporous hydrogels formed at subzero temperature by polymerization of monomers or by gelation of polymeric precursors by the phenomenon of cryogelation [4–6]. During this process, the monomeric or polymeric precursors are dissolved in solvent such as deionized water followed by the addition of crosslinkers. Whole mixture is then incubated under frozen conditions immediately. During the formation of cryogels at subzero temperature, the solvent freezes which results in the formation of ice crystals (which act as porogens). These ice crystals further grow and interconnect to each other. Some part of solvent or solute molecules present in the solution remains unfrozen (known as unfrozen liquid microphase). Polymeric reactions take place in liquid microphase where these solute precursors polymerize and get crosslinked which leads to the formation of gel. After thawing of cryogels, these ice crystals melt leaving the void spaces which results in an interconnected polymeric network [3]. These matrices facilitate the unhindered diffusion of solutes and nutrients due to the presence of interconnected macropores [7–10]. These matrices have pore size up to 200 *μ*m which depends on the polymeric precursor initial concentration, their physicochemical properties, and the freezing conditions [11]. Cryogelation technique has an advantage over other fabrication techniques as cryogels can be fabricated in different sizes and formats like disc, sheets, or monoliths with varying dimensions. Interconnected macroporous architecture and interpenetrating network (IPN) makes cryogels appropriate scaffolds for the biomedical and biotechnological applications [3, 12–17]. Cryogel matrices have shown applicability in cell chromatography, bioreactors for monoclonal antibody production, affinity chromatographic separations, cell separation, and tissue engineering [18–21].

In this research work, we have fabricated the scaffolds using the combination of natural polymers by cryogelation technique. The natural polymers carrageenan and gelatin were selected for the fabrication of 3D cryogel matrices. Carrageenan sodium salt is a hydrophilic polysaccharide that exists in nature as an extracellular matrix material in species of red seaweeds. It has a basic linear primary structure based on a repeated disaccharide unit of *α* (1-3) D galactose and *β*(1-4)3,6-anhydro-D galactose and contains sulphate group per disaccharide unit. It has been reported to have an anticoagulant effect due to sulphonyl groups. Kappa carrageenan-gelatin scaffold synthesized by freeze drying technique has been proven to improve blood compatibility [22]. Carrageenan sodium salt has shown applications in the production of macroporous composites for bone tissue engineering [23] and wound dressing [24]. It is also being used for microencapsulation and immobilization of drug [25, 26]. Other natural polymer, that is, gelatin is a biocompatible, biodegradable, nonimmunogenic, adhesive polymeric protein derived from thermal denaturation of collagen and isolated or extracted from bones and fish skin. It is widely used as a scaffolding material for tissue engineering as well as in biomedical applications such as wound dressing and as an adhesive or absorbent pad for surgical use [3, 12, 16, 27]. Both these polymers after the fabrication of scaffolds showed good mechanical properties, retained shape after deformation, and provided a microenvironment for the proliferation of cells which are the perquisites of a good biomaterial for biomedical and tissue engineering applications. 

The purpose of the present study is to synthesize supermacroporous carrageenan-gelatin cryogels using cryogelation technology for their use in biomedical and other bioengineering applications. These cryogel scaffolds were crosslinked using different concentrations of glutaraldehyde and 1-ethyl-3-[3-dimethylaminopropyl]carbodiimide hydrochloride-N-hydroxysuccinimide (EDC-NHS) and were characterized chemically, physically, and mechanically in terms of pore volume, porosity, cyclic swelling kinetics, swelling ratio, flow characteristics, density, X-ray diffraction, thermogravimetric analysis, Fourier transform infrared, *in vitro* degradation rate, unconfined compression, and rheological analysis. Biocompatibility was analyzed by the manifestation of cell proliferation and secretion of ECM by growing the fibroblast cells (Cos-7) on the synthesized cryogel matrices.

## 2. Materials and Methods

### 2.1. Materials

Gelatin (from cold water fish skin; M.W: 60,000), 3-(4,5 dimethylthiazol-2-yl)-2,5-diphenyl tetrazolium bromide (MTT, 98%) reagent, 1-ethyl-3-(3-dimethylaminopropyl) carbodiimide (EDC), propidium iodide (PI), Dulbecco's modified eagle's medium (DMEM), antibiotic-antimycotic solution, 0.25% trypsin, and phosphate-buffered saline (PBS) were purchased from Sigma Chemical Co. (St. Louis, MO, USA). Fetal bovine serum (FBS) was purchased from Hyclone (UT, USA). *N*-Hydroxysuccinimide (NHS), carrageenan sodium salt (Irish Moss), and glutaraldehyde solution (25%) were bought from S.D. fine chemicals (Mumbai, India). Dimethyl sulfoxide (DMSO) was purchased from Qualigens fine chemicals (Mumbai, India). Fibroblast (Cos-7) cell line was procured from National Centre for Cell Science (Pune, India). All the other chemicals used in the study were of analytical grade. 

### 2.2. Synthesis of Supermacroporous Carrageenan-Gelatin Cryogels

In this study a combination of two natural polymers carrageenan and gelatin was used for the preparation of gel matrices by the process of cryogelation with two different crosslinking agents, that is, glutaraldehyde and 1-ethyl-3-[3-dimethylaminopropyl] carbodiimide hydrochloride-*N*-hydroxysuccinimide (EDC-NHS). During optimization different concentrations of carrageenan : gelatin were tried for the fabrication of scaffolds. Different ratios of carrageenan : gelatin used were in the range of 0.5% to 2% : 2% to 3% and crosslinkers, that is, glutaraldehyde in the range of 150 to 250 *μ*L and EDC-NHS from 15 mg–5 mg to 45 mg–15 mg. For the fabrication of carrageenan-gelatin cryogel (CGG) using glutaraldehyde as a crosslinker, 150 mg of carrageenan was dissolved in 10 mL of deionized water whereas in the case of carrageenan-gelatin cryogel (CGE) with EDC-NHS as a crosslinker 100 mg of carrageenan was dissolved in 10 mL of deionized water. Corresponding solutions for both the polymers were made by boiling the mixture at 60 to 70°C for half an hour till the solutions become clear followed by cooling at room temperature. To the above mixture 300 mg of gelatin was added in both the tubes having carrageenan solution followed by the addition of 160 *μ*L of glutaraldehyde from the stock solution (25% v/v) glutaraldehyde for CGG and EDC-NHS (45 mg–15 mg) per 10 mL of solution as crosslinking agent for CGE. Carrageenan-gelatin solutions were thoroughly mixed on a vortex. The resultant solution was then incubated at −12°C for 16 h in cryostat (Seelbach, Germany). After the incubation gels were removed and thawed in deionized water at room temperature. After thawing, carrageenan-gelatin cryogels were lyophilized at −57°C overnight in lyophilizer (Martin Christ GmbH, Germany) which were then used for further experiments.

### 2.3. Microstructure Analysis (SEM)

The morphology of composite cryogel samples was investigated using scanning electron microscope (SEM, FEI Quanta 200). All the scaffolds; that is, carrageenan-gelatin crosslinked with glutaraldehyde and carrageenan-gelatin cryogels crosslinked with EDC-NHS were lyophilized at −57°C overnight. Samples were then gold coated using a sputter coater (Vacuum Tech, Bangalore, India). Surface of these coated scaffolds was then scanned by electron microscope operated at high vacuum and high voltage of 20 kV.

### 2.4. Determination of the Flow Characteristics

The flow rate of carrageenan-gelatin cryogels synthesized with two different crosslinkers, that is, glutaraldehyde and EDC-NHS was evaluated by allowing the liquid such as deionized water to pass through the cryogel matrix. Cryogel scaffolds of specific dimensions (diameter: 13 mm and thickness: 20 mm) were inserted in plastic syringe which was connected with peristaltic pump. Flow rate across all these cryogel samples was examined by passing deionized water under pressure at a controlled speed up to the flow at which cryogel does not show any backpressure. In case of control sample flow rate was determined without connecting cryogel samples in between the flow path [17].

### 2.5. Pore Volume, Porosity, and Density Analysis

Pore volume which is a ratio of porous materials air volume to porous materials total volume was calculated by cyclohexane uptake method [3]. Cryogel sections of 5 mm thickness and 13 mm diameter were used and immersed in cyclohexane at room temperature for 1 h. Pore volume of carrageenan-gelatin cryogels was determined by

(1) $$\begin{array}{c} \text{Pore}\;\text{volume}=\frac{M\left(\text{swollen}\;\text{gel}\right)-M\left(\text{dried}\;\text{gel}\right)}{M\left(\text{dried}\;\text{gel}\right)}. \end{array}$$

Porosity which designates the presence of overall pores in the carrageenan-gelatin cryogel samples was determined using specific gravity bottle with the help of Archimedes's principle. Cryogel sections were submerged under deionized water in a specific gravity bottle and the submerged mass of the cryogel sample was recorded [14]. The cryogel was then taken out from specific gravity bottle having deionized water and swollen or wet mass of cryogel was recorded. All these cryogel sections used for determination of porosity were in triplicates and percent porosity was calculated as

(2) $$\begin{array}{c} \%\;\text{Porosity}=\frac{\;M_{s}-M_{d}}{M_{s}-M_{\text{sub}}}\times 100, \end{array}$$

where *M*_*s*_ is wet mass of the cryogel saturated with deionized water, *M*_*d*_ is the dry mass of the cryogel, and *M*_sub_ is the submerged mass of the cryogel.

The densities in gram per cubic centimetre of dry and wet cryogel samples were determined [12] by the ratio of dry weight as well as wet weight of the samples to their volume, respectively, by

(3) $$\begin{array}{c} d=\frac{W}{\pi }\times {\left(\frac{D}{2}\right)}^{2}\times H, \end{array}$$

where *d* is the apparent density, *W* is weight of cryogel sample in grams, *D* is diameter of sample in centimetre, and *H* is thickness of sample in centimetre.

### 2.6. Cyclic Swelling Kinetics and Swelling Ratio

The solvent uptake capacity of a porous material with respect to time at a particular temperature was determined according to the conventional gravimetric procedure [28]. Swelling kinetics is the measure of solvent uptake capacity by a material. Swelling and deswelling of the cryogel samples were done up to five cycles for the detection of any changes in the behavior of the cryogels from initial to the final cycle [15]. Initially, the dry weights of cryogel sections (diameter: 13 mm and thickness: 5 mm) were taken in triplicates and incubated in 0.1 M PBS (pH 7.4) for a particular time duration. The wet weight was recorded at specific time intervals till the samples were equilibrated with the PBS. After equilibration cryogel sections were completely dried at 60°C in the oven and dry weight was recorded. Cryogels were again subjected to second swelling cycle. Similar deswelling and swelling cycles were repeated up to five cycles. The percent retention of water (Wr) and weight-swelling ratio were determined by

(4) $$\begin{array}{c} W_{u}=\frac{W_{t}-W_{g}}{W_{e}}\times 100, \end{array}$$

where, *W*_*u*_ is the solvent uptake capacity, *W*_*t*_ the weight at regular time interval, *W*_*g*_ the weight of dry cryogel and *W*_*e*_ the weight of water in swollen gels at swelling equilibrium at a particular temperature.

The weight-swelling ratio was taken as a parameter to calculate solvent absorption capacity. It was calculated as:

(5) $$\begin{array}{c} \text{SR}=\frac{W_{s}-W_{d}}{W_{d}}, \end{array}$$

where SR is the swelling ratio, *W*_*s*_ is the weight of swollen gel, and *W*_*d*_ is the weight of dry gel.

### 2.7. Fourier Transform Infrared (FTIR) Spectroscopy

Carrageenan-gelatin cryogel samples were prepared in powder form by flash dipping in liquid nitrogen. Different functional groups as well as the bond linkage were analyzed by the Fourier transform infrared analysis (FTIR). Infrared spectra of specimen powders were recorded on a Perkin-Elmer 1000 paragon spectrometer. 

### 2.8. X-Ray Diffraction (XRD) Analysis

X-ray diffractometer (Mini Flex II, Rigaku, Japan) employing Cu-K*α* (0.15405 nm) radiation was used to investigate the crystallinity and phase content of the carrageenan-gelatin scaffolds prepared in powder form with the help of liquid nitrogen. Data was collected from 3 to 80 degrees with a sampling width of 0.01 inch and a scanning speed of 3 degree/min [29].

### 2.9. Thermogravimetric Analysis (TGA)

Thermogravimetric analysis (TGA) was carried out on the cryogel samples in powder form prepared by flash dipping in liquid nitrogen. Samples were kept in a platinum pan using a DTG-60 (Shimadzu, Japan). The experiments were conducted under inert atmosphere (nitrogen gas) between temperature range of 30 to 750°C at a heating rate of 10°C/min [29].

### 2.10. Unconfined Compression Analysis

The compressive modulus of carrageenan-gelatin cryogels was determined using dynamic mechanical analyzer (DMA) Zwick/Roell Z010. Cryogel samples of dimensions of 13 mm thickness and 13 mm diameter were saturated with PBS (pH 7.4) and were analyzed for unconfined compression. Saturated cryogel samples were uniaxially compressed up to 90% of their original length under a load cell of 500 N for dry cryogel samples and 20 N for wet samples at the displacement rate of 1 mm/min by placing them between two arms of load frame at room temperature. The applied force and change in column at room temperature were recorded. Each cryogel sample was analyzed in triplicates to reduce the error. Average unconfined compressive modulus of all different cryogel samples was calculated from the stress (kPa) versus strain (%) graph [15].

### 2.11. *In Vitro* Degradation Analysis

For the analysis of *in vitro* degradation dry samples of carrageenan-gelatin cryogels were prepared using two different crosslinkers, that is, glutaraldehyde and EDC-NHS. Samples were analyzed in triplicates to reduce the manual error while handling the samples. All the samples were weighed and sterilized by treating them with a series of ethanol (20–100%) for 10–15 min in each concentration. Ethanol-sterilized cryogel samples were then dried overnight in oven and transferred to 50 mL centrifuge tubes containing sterile 0.1 M PBS (pH 7.4). Samples were then incubated in a water bath at 37°C for 4 weeks under sterile conditions. After every week, samples were removed and washed with deionized water twice to remove the digested polymer. Samples were then dried overnight in oven at 60°C and dry weight was recorded [15]. The degree of degradation was calculated as

(6) $$\begin{array}{c} \text{D}\cdot \text{D}\;\%=\frac{W_{I}-W_{F}}{W_{F}}\times 100, \end{array}$$

where D · D is degree of degradation, *W*_*I*_ is initial dry weight of sample before incubation and *W*_*F*_ is final dry weight of sample after incubation. Morphological changes in degraded sample were analyzed.

### 2.12. Rheology Analysis for Carrageenan-Gelatin Cryogels

Rheology helps in analyzing the flow and deformation behaviour of polymeric material and its internal structure at given conditions. Cryogel sections of diameter 8 mm and thickness 5 mm were analyzed at a force of 1 N s^−1^ for 15 min at 37°C. All cryogel sections were kept on the holder plate of diameter 12.5 mm and checked for phase difference under dry and wet states. For testing in dry state, cryogel samples were dried by lyophilizing them. Samples were also analyzed under wet state which was done by saturating them with PBS before the study [30].

Viscoelastic materials have properties that they manifest the combination of two attributes that is, storage and loss modulus. For the determination of storage or elastic modulus (*G*′) and loss modulus (*G*′′), oscillatory logarithmic sweep at a frequency of 1 Hz and a strain of 0.1% was used. Storage modulus was termed as the solid behavior of the material as it dissipate all the energy obtained from deformation and when the deforming stress is removed, none of the energy is recovered. The elastic nature will develop in phase stress with strain:

(7) $$\begin{array}{c} G^{\prime }=\mathrm{Cos}\delta \left(\frac{\sigma_{0}}{\gamma }\right), \end{array}$$

loss modulus (*G*′′) represented the liquid behavior and all the energy is retained within material and when the stress is removed, energy is recoverable. It gives information about dissipation (viscous) of the flow:

(8) $$\begin{array}{c} G^{\prime \prime }=\mathrm{Sin}\delta \left(\frac{\sigma_{0}}{\gamma }\right), \end{array}$$

where *σ*_0_ is the shear stress, *γ* is the amplitude of the strain response, and *δ* is the phase angle (angle between the applied and measured stress response).

### 2.13. Microscopic Analysis of Cell-Seeded Scaffolds (SEM and Propidium Iodide (PI) Staining)

To analyze the interaction of cells with the cryogel matrices fibroblast cells (Cos-7) were seeded on cryogel sections. Samples were analyzed by SEM on 2nd and 7th days of the experiment. On the day of analysis, media were removed from the wells followed by washing of seeded scaffolds with 0.1 M cold PBS (pH 7.4). Cells were fixed to the surface of cryogel matrix by treating with 2.5% glutaraldehyde for 6 h at 4°C. After fixation cryogel scaffolds were dehydrated by treating them with gradient concentration of ethanol (20–100%) for 15 min in each concentration. Ethanol was removed from the scaffolds by thorough washing with 0.1 M cold PBS (pH 7.4). Cryogel samples were then dried under vacuum for the complete removal of moisture. To analyze the samples under SEM surface was coated with gold using sputter gold coater [12]. To observe the interaction of cryogel matrix with the fibroblast (Cos-7) cells, PI nuclear stain was also used. Cryogel monoliths were cut into the sections of thickness 200 *μ*m using microtome (Microm HM 560 Cryo Star, Thermo). These cryogel sections were then sterilized by treating them with series of ethanol (20–100%) concentrations. After sterilization all cryogel sections were allowed to saturate with complete media at 37°C. Fibroblast cells (Cos-7) were seeded at a density of 1 × 10^5^ cells/mL and plates were incubated at 37°C for specific duration of time. PI staining was done after 5th day of cell culture to see the cell adherence and proliferation. The complete media were removed from the wells having cell seeded sections. Sections were then gently washed with 0.1 M cold PBS (pH 7.4) thrice. Seeded cryogel sections were fixed with 4% paraformaldehyde for 2 min at room temp. Cells were then permeabilized with permeabilizing solution (containing triton X-100) for 5 min at 4°C followed by washing three times with 0.1 M PBS. PI nuclear stain (10 *μ*g/mL working solution) was then added followed by the incubation of sections for 30 s at room temperature. After incubation sections were gently washed with 0.1 M cold PBS three times. Sections were observed under the fluorescence microscope using excitation and emission wavelength of PI at 536 nm and 617 nm, respectively. Fluorescent images were taken by selecting three random microscopic fields.

### 2.14. *In Vitro* Biocompatibility Analysis of Scaffolds (MTT Assay)

To evaluate the biocompatibility of cryogel matrices, proliferation of a representative cell line, that is, Cos-7 was analyzed. Carrageenan-gelatin cryogel scaffolds of diameter 8 mm and thickness 2 mm were taken in triplicates for the analysis of cytotoxicity and cell proliferation by 3-(4,5-dimethylthiazol-2-yl)-2,5-diphenyltetrazolium bromide (MTT) assay [31]. Cryogel scaffolds were sterilized by treating them with a series of ethanol (20–100%) for 10–15 min in each concentration, followed by washing with 0.1 M cold PBS (pH 7.4) three times. Cryogel scaffolds were then placed in 48-well plate and saturated with complete DMEM at 37°C for 2-3 h. To study the competency of all cryogels as a scaffold for cell adhesion fibroblasts cells (Cos-7) were used and seeded directly onto saturated cryogel sections at a seeding density of 1 × 10^5^ cells/mL. As control, cells were seeded on tissue culture-treated wells which represented a two-dimensional (2-D) system. Control wells were treated in the same way as that of test wells. Both test and control plates were incubated at 37°C in an incubator with humidified 5% CO_2_ for 15 days. Media were changed after every 2 days up to 15 days of cell culture experiment. MTT assay was performed after every alternate day. On the day of assay media were removed from the wells (test and control) followed by gentle washing with 0.1 M cold PBS (pH 7.4). MTT working solution was prepared by dissolving 0.5 mg MTT per mL of plain DMEM. From this solution, 250 *μ*L was added to the control and test wells; plates were incubated for 4-5 h at 37°C with 5% CO_2_. After the incubation, MTT reagent was gently aspirated without disturbing the scaffold followed by the addition of 750 *μ*L dimethyl sulfoxide (DMSO) in wells. The plates were again incubated for 10–15 min at 37°C to dissolve the reduced MTT. The developed colour was measured at 570 nm spectrophotometrically to calculate the relative viability of fibroblast cells (Cos-7) on the scaffolds which in turn illustrates the biocompatibility of the material [12].

## 3. Results

### 3.1. Fabrication of Carrageenan-Gelatin Cryogels

**Figure 1:**
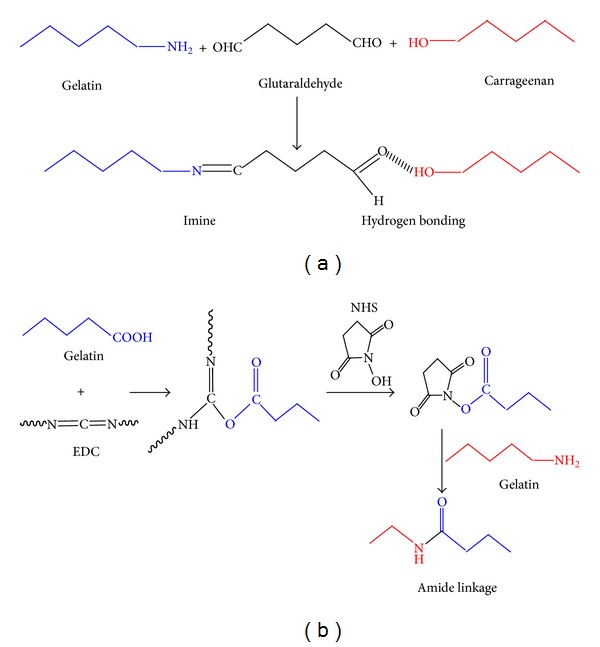
Schematic representation of (a) crosslinking between carrageenan and gelatin polymer precursors using glutaraldehyde as a crosslinker and (b) crosslinking between gelatin polymer precursors present in carrageenan-gelatin cryogel using EDC-NHS as a crosslinker.

**Figure 2:**
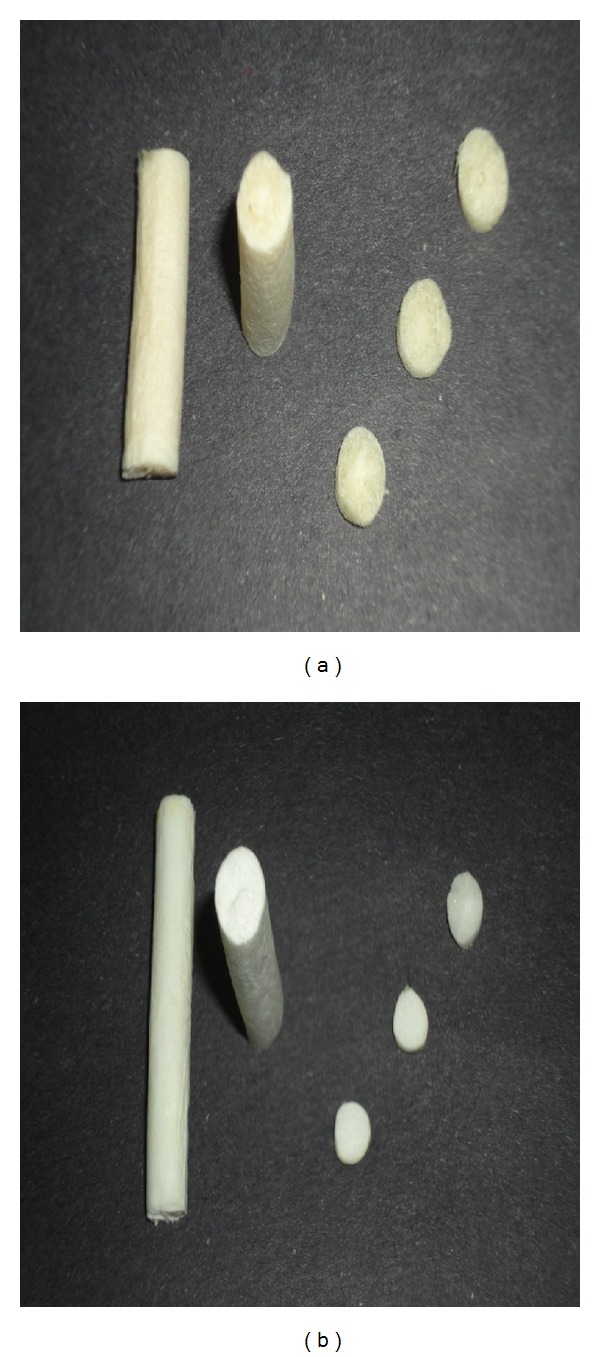
The digital images of different format like in monolith and disc forms of carrageenan-gelatin cryogels (a) CGG with glutaraldehyde as a crosslinker and (b) CGE with EDC-NHS as a crosslinker.

All cryogels CGG and CGE were synthesized using glutaraldehyde and EDC-NHS as crosslinking agents. Probable crosslinking mechanism of synthesis of cryogel matrices is depicted in Figure 1. Carrageenan being a sulphated polysaccharide lacks the functional groups required for crosslinking with either glutaraldehyde or EDC/NHS; therefore an interpenetrating network (IPN) [16] of carrageenan-gelatin is synthesized. Moreover, gelatin cryogels have already been synthesized and characterized previously [17]. *In vitro* studies on these cryogels have been done and similar trend in cell proliferation has been observed (unpublished data). Physical appearance of synthesized carrageenan-gelatin cryogels (glutaraldehyde as crosslinker) was observed as a light yellow in colour while the cryogels synthesized using EDC-NHS as a crosslinker appeared white in colour (Figure 2). For the fabrication of cryogel matrices the concentrations of crosslinkers and polymers were optimized in order to obtain an ideal gel for tissue engineering applications. In CGG final optimized concentration for carrageenan and gelatin was established to 15 mg/mL and 30 mg/mL, respectively; concentration of glutaraldehyde was optimized to 16 *μ*L/mL of 25% v/v of glutaraldehyde stock solution. In case of other matrix CGE, final optimized concentration for carrageenan and gelatin was found to be 10 mg/mL and 30 mg/mL, respectively, while the concentration of EDC-NHS was optimized to 4.5 mg–1.5 mg per mL of gel solution.

### 3.2. Microstructure Analysis (SEM)

**Figure 3:**
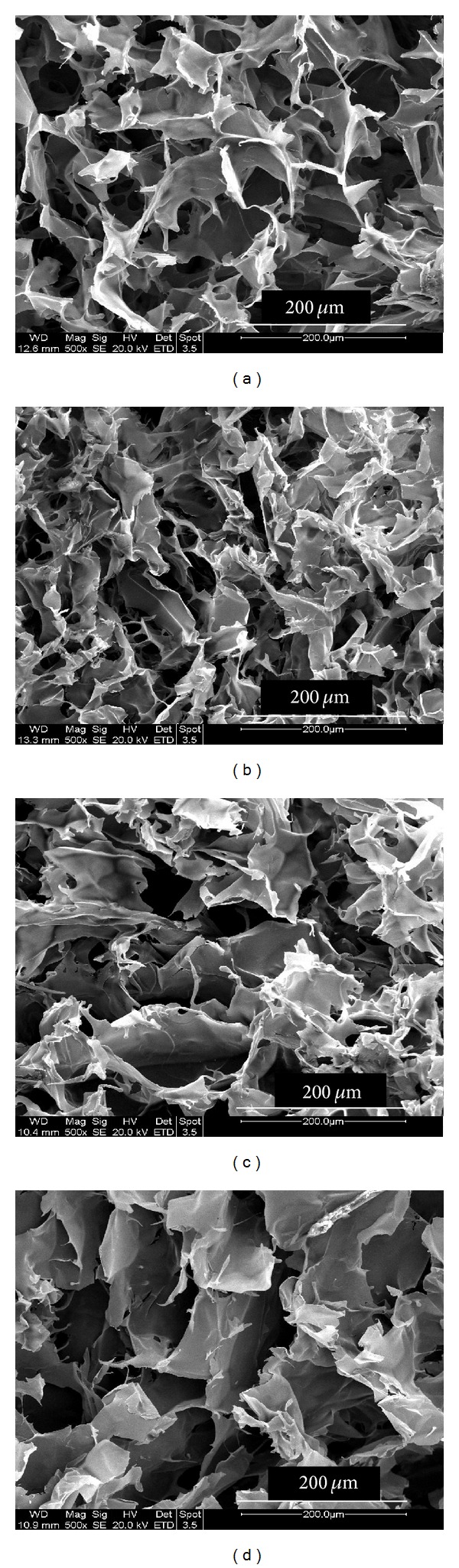
Scanning electron microscopic (SEM) images of both horizontal cross sections (a) and (b) and lateral cross sections (c) and (d) of macroporous structure of synthesized carrageenan-gelatin cryogels. (a) and (c) are CGG cryogels having glutaraldehyde as a crosslinker. (b) and (d) are CGE cryogels with EDC-NHS as a crosslinker.

Microstructure analysis of two different cryogels, that is, CGG and CGE was done by taking lateral and horizontal cross sections (Figure 3). Average pore size of these cryogels synthesized using two different crosslinkers such as glutaraldehyde and EDC-NHS was almost similar and was found to be in the range of 60–100 *μ*m. SEM analysis revealed that scaffolds possess large and interconnected pores which were generated by the process of cryogelation. In both types of cryogel matrices pores were observed to be arranged in a structured layout.

### 3.3. Flow Rate Analysis

The flow rate of synthesized cryogels, that is, CGG, CGE was measured by allowing the deionized water to flow across the cryogel samples using peristaltic pump (Table 1). All the synthesized cryogels showed flow rate in the range of 2–4 mL/min without any backpressure. Flow rate which indicates the presence of interconnected porous network in the gel is also confirmed by SEM as mentioned in the previous section.

### 3.4. Pore Volume, Porosity and Density Analysis of Cryogel Matrices

Pore volume and porosity of the synthesized cryogels were analyzed by using cyclohexane uptake capacity and through Archimedes's principle, respectively. Cryogel matrices, that is, CGG synthesized with glutaraldehyde crosslinker showed pore volume of 12.78 ± 0.96 while CGE with EDC-NHS showed 14.3 ± 0.52. Porosity in both cases was observed in the range of 75 to 85% (Table 1). Densities of dry and wet carrageenan-gelatin (CGG and CGE) cryogels were calculated by measuring their weight and dimensions. Study was conducted in triplicates to avoid the manual error and details are shown in (Table 1).

### 3.5. Cyclic Swelling Kinetics and Relative Swelling Ratio

**Figure 4:**
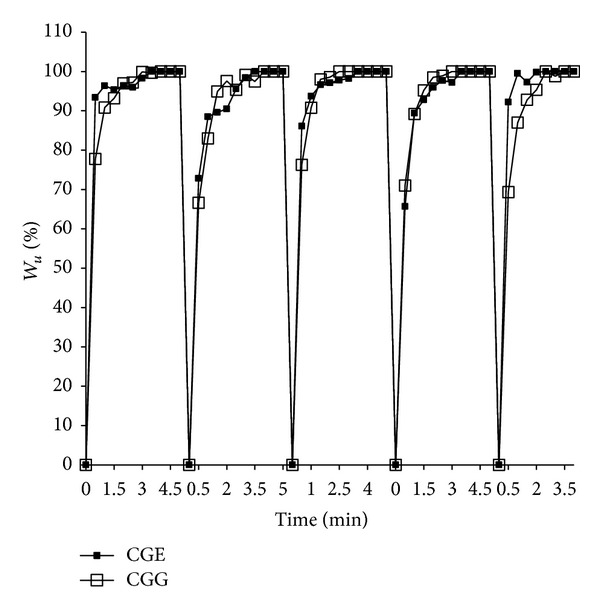
Cyclic swelling kinetics of carrageenan-gelatin cryogels CGG (open squares), CGE (filled squares).

Cyclic swelling kinetics of cryogels was determined by the conventional gravimetric method. The cryogels were synthesized by two different crosslinkers, that is, glutaraldehyde and EDC-NHS. Swelling and deswelling were done for all types of cryogels. It was observed that cryogels swelled up to 90% within 1 min and attained equilibrium within 3 min. During the experiment constant behavior of cryogels was observed from the first cycle to fifth cycle. Swelling kinetics and relative swelling of the cryogels were obtained from the graph in Figure 4. Average swelling ratio was also calculated for these cryogels and is listed in Table 1.

### 3.6. Fourier Transform Infrared (FTIR)

**Figure 5:**
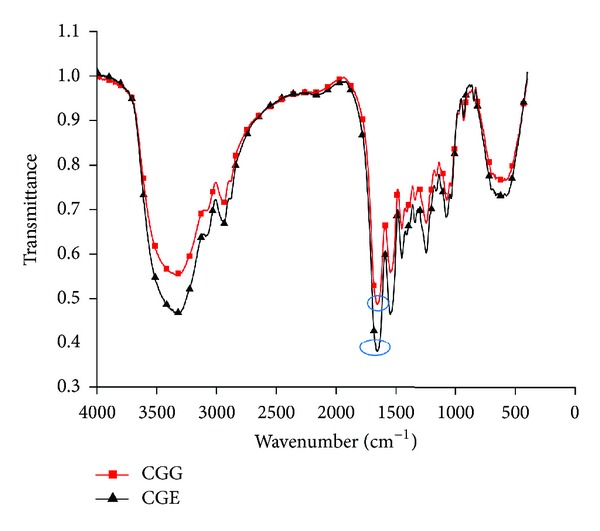
FTIR spectrograph of carrageenan-gelatin cryogels using glutaraldehyde and EDC-NHS as crosslinkers showing encircled prominent peak for amide linkage and imine formation.

The presence of functional groups and linkage which occur between the polymers used for the synthesis of cryogels was confirmed by the Fourier transform infrared (FTIR) spectroscopy. Distinguishable absorption bands peak of the amide linkage was observed in the range of 1630–1680 cm^−1^. This amide linkage peak was observed in both types of cryogels, that is, CGG and CGE (Figure 5). One more peak of imine formation was observed in case of CGG in approximately the same range as that of amide. Few overlapping peaks were also obtained due to some similar functional groups present in the polymers.

### 3.7. X-Ray Diffraction (XRD) Analysis

**Figure 6:**
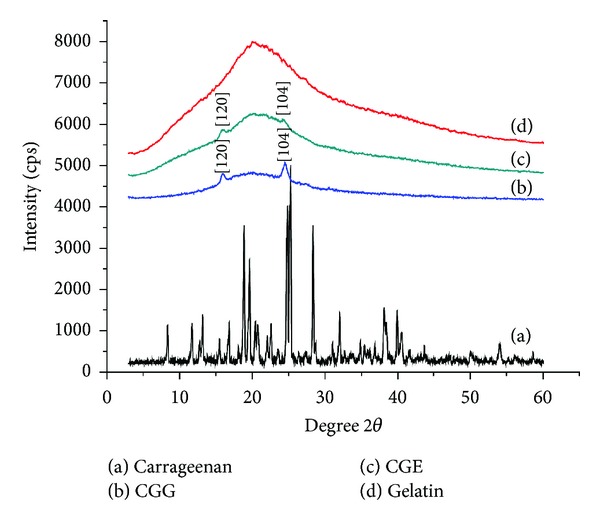
X-ray diffraction analysis of carrageenan-gelatin cryogels CGG (b) CGE (c) and compared with neat carrageenan (a) and neat gelatin polymer (d).

X-ray diffraction analysis of blend of carrageenan-gelatin was done and compared with the polymers used in the blend which showed some crystalline peaks. Neat carrageenan polymer gave some characteristic peaks in the diffraction pattern which were found similar to the peaks obtained from carrageenan-gelatin blend solution at 2*θ* = 16.09°, 24.74° corresponding to [120], [104] diffraction planes, respectively, and neat gelatin present in the blend solution did not show any peaks (Figure 6).

### 3.8. Thermogravimetric Analysis (TGA)

**Figure 7:**
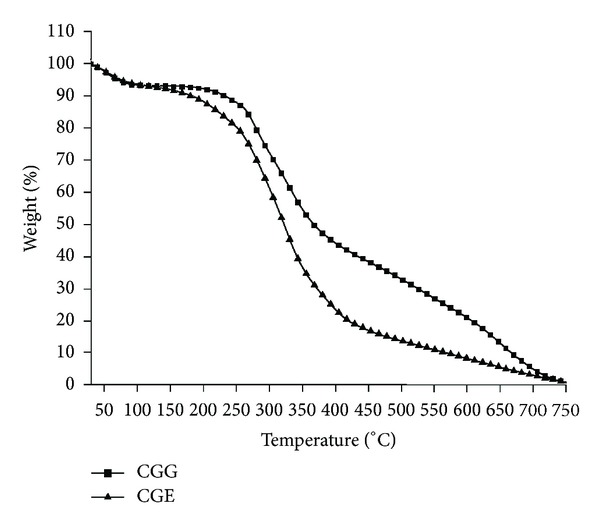
Thermogravimetric analysis of carrageenan-gelatin cryogels using glutaraldehyde and EDC-NHS as a crosslinker. CGG (filled squares) and CGE (filled triangles).

The thermal decomposition of the scaffold materials was studied using TGA. Powder forms of carrageenan-gelatin cryogels with two different crosslinkers were heated from 30 to 750°C under inert atmosphere. First 5% and 10% weight losses due to moisture were observed in both cryogels (Figure 7) and values are shown in Table 1.

### 3.9. Unconfined Compression Analysis

**Figure 8:**
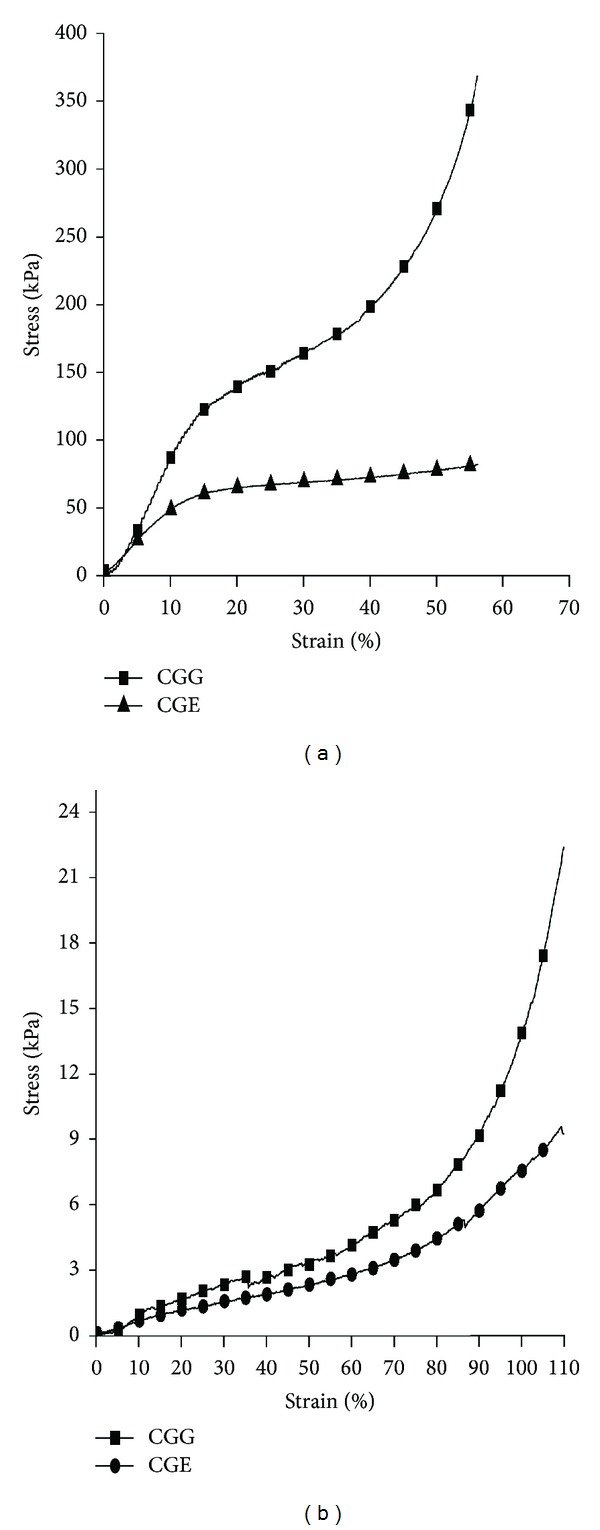
Unconfined compression stress-strain curve of carrageenan-gelatin cryogels (CGG, CGE) given with 500 N and 20 N load cell under displacement control at the rate of 1 mm/min for dry and wet cryogel samples, respectively. (a) Dry cryogel section of CGG (filled squares) and CGE (filled triangles). (b) Wet cryogel section of CGG (filled squares) and CGE (filled circles).

The synthesized scaffolds should maintain their mechanical integrity or should provide mechanical strength during any mechanical strain applications. Compression property of all carrageenan-gelatin was determined by the uniaxial stress exerted on the cryogels. Unconfined compression analysis was done on the synthesized cryogels which gives a relationship between stress exerted and strain experienced by the cryogel to obtain stress-strain data. Young's modulus was obtained for the estimation of elasticity and mechanical strength of the cryogels from the slope of the curve between stress versus strain graphs (Figure 8). Young's modulus for cryogel sections in wet condition was found less as compared to the cryogel sections in dry condition shown in Table 1. However, the cryogels synthesized from two different crosslinkers were compressed up to 90% of their original length and there was no permanent deformation or cracks observed in both cryogels present in both the wet and dry conditions. 

### 3.10. *In Vitro* Degradation Analysis

The degree of degradation of all cryogels synthesized from two different crosslinkers, that is, glutaraldehyde and EDC-NHS under sterile conditions was determined by the change in dry weight of the test samples. The degradation characteristics of all cryogels were examined after 4 weeks of incubation in 0.1 M PBS (pH 7.4) at 37°C under sterile conditions. Carrageenan-gelatin cryogel synthesized by using glutaraldehyde as a crosslinker (CGG) showed more degree of degradation, that is, 50.55 ± 5.49% as compared to carrageenan-gelatin cryogel (CGE) with EDC-NHS which showed the degradation of 34.17 ± 2.52% shown in Table 1. Degraded samples exhibit a prominent weight and size reduction in comparison to the normal samples.

### 3.11. Rheological Analysis of Carrageenan-Gelatin Cryogels

**Figure 9:**
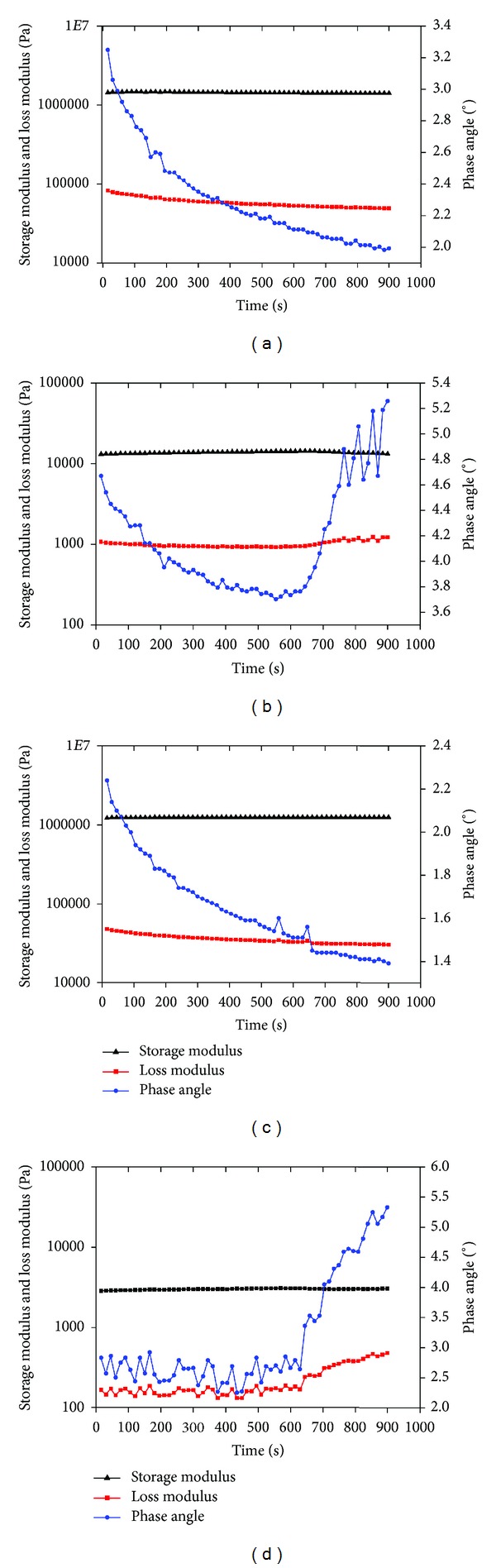
Rheological analysis of carrageenan-gelatin cryogels, CGG in dry (a) and wet (b) forms, CGE in dry (c) and wet (d) form at 37°C showing storage modulus, loss modulus and phase angle parameters variation with time.

In order to confirm the utility of the cryogels for tissue engineering applications rheological analysis was done. This analysis was done to determine the viscoelastic behaviour of the cryogels under a specific temperature. This study was conducted by taking three parameters into consideration, that is, storage modulus, loss modulus, and phase angle. These cryogels showed different storage and loss modulus with constant temperature in both dry and wet states. Dry cryogel sections showed more storage (*G*′) and loss modulus (*G*′′) with different range of phase angle as compared to wet cryogel sections. Storage modulus in both the cryogels, that is, CGG and CGE in dry state was more than the cryogels in wet state and was in the range of 12 × 10^6^–14 × 10^6^ Pa. Storage modulus in wet condition for CGG was found to be more than CGE and it was in the range of 13 × 10^3^–14 × 10^3^ Pa whereas CGE was observed to be 2 × 10^3^–3 × 10^3^ Pa. Phase angle decreases in both the cryogels present in dry condition, while in case of CGG phase angle was in the range of 3.3° to 2° (approximately) and for CGE, range was 2.3° to 1.35°. Phase angle in wet conditions for both the cryogels was varying but increased from 4.7° and 5.3° in the case of CGG whereas in CGE phase angle variation was more in between 2.5° to 3° but then it increased to 5.4° (Figures 9(a), 9(b), 9(c), and 9(d)).

### 3.12. Microscopic Analysis of Cell-Seeded Scaffolds (SEM and Propidium Iodide (PI) Staining)

**Figure 10:**
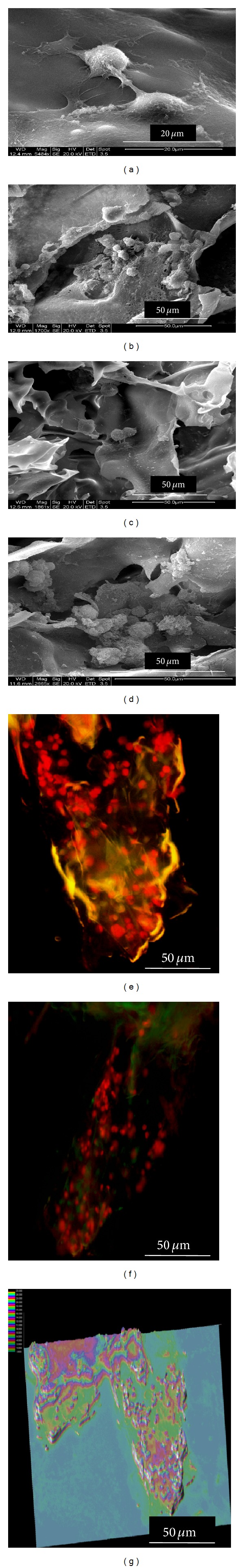
(a) Scanning electron micrographs of fibroblasts cells (Cos-7) in dividing stage on CGE cryogel matrix after 2nd day of culture at 5484x magnification, (b) Cos-7 on CGE after 7th day of culture at 1700x magnification, (c) Cos-7 cells on CGG after 2nd day of culture at 1861x magnification, and (d) Cos-7 on CGG after 7th day of culture with more ECM on matrix at 2865x magnification. (e), (f), (g) Fluorescence microscope images of fibroblast Cos-7 cells seeded on a 200 *μ*m carrageenan-gelatin cryogel section analyzed by nuclear staining using PI stain. (e) PI staining image of cryogel with cells after 5th day of culture. (f) Composite image of cryogel with cells after 5th day of culture. (g) Surface plot of carrageenan-gelatin cryogel with cells.

*In vitro* cell culture experiment was performed to observe the interaction between the cells and scaffold material. Here, fibroblast (Cos-7) was used as a model cell line to test the material ability for cell adhesion. Cells were seeded at a density of 1 × 10^5^ cells per mL (250 *μ*L per well in 48 well plate) on all sterilized cryogels. Seeded scaffolds were incubated for a definite period of time under favorable conditions. Cryogel scaffolds without cells were taken as control. After 2nd day and 7th day cell adherence, growth, and proliferation were examined by SEM. The analysis (Figures 10(a), 10(b), 10(c), and 10(d)) showed that cells have attached to the surface of the matrix and have produced extracellular matrix. SEM analysis also shows the presence of more number of cells on the 7th day than on 2nd day, which indicates that cells are proliferating on the cryogel matrix together with the production of ECM. SEM analysis also shows the presence of dividing fibroblast (Figure 10(a)) on the gel matrix which indicates that matrix is supporting the growth and proliferation of the fibroblast cells. Adherence and proliferation of fibroblast cells (Cos-7) on the synthesized carrageenan-gelatin cryogels were also confirmed by seeding them on 200 *μ*m sections. Seeded sections were stained with PI to observe the nuclei of the cells. The PI staining has been done after 5th day where one could observe the presence of cells on the sections (Figures 10(e), 10(f), and 10(g)). Bright red fluorescence under TRITC filter which has an excitation wavelength of 536 nm and an emission wavelength of 617 nm is an indication that cryogels support the adherence of the fibroblast cells on them.

### 3.13. *In Vitro* Biocompatibility (MTT Assay)

**Figure 11:**
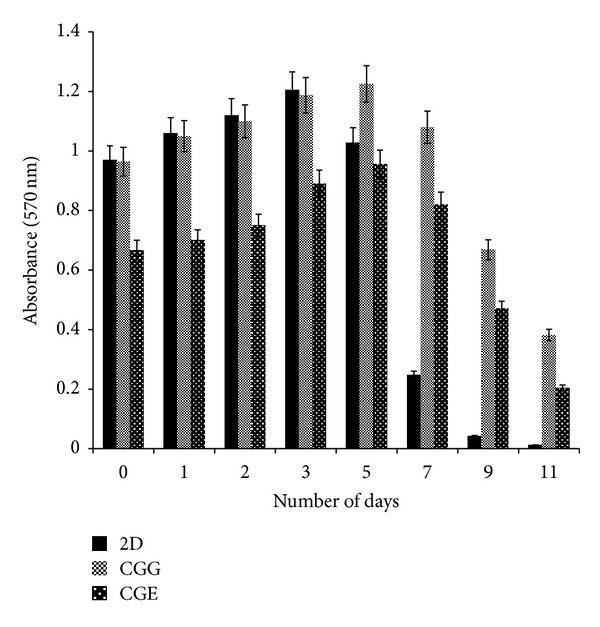
The relative viability of fibroblast (Cos-7) cells as determined by MTT assay. Cells were grown up to 10 days on polystyrene-coated tissue culture plate well used as a control (solid bar); carrageenan-gelatin synthesized with two different crosslinkers like glutaraldehyde (CGG—striped bar) and EDC-NHS (CGE—dotted bar). The absorbance of blue formazan was measured at 570 nm at different time intervals up to ten days of culture.

The viability and proliferation of fibroblast cells on the cryogel matrices were determined by 3-(4,5-dimethylthiazol-2-yl)-2,5-diphenyltetrazolium bromide, a tetrazole (MTT) assay. This assay is based on the principle that viable proliferating cells will convert tetrazolium salt, that is, MTT to an insoluble product, purple formazan. These formazan crystals can be further solubilized in an organic solvent like dimethylsulfoxide (DMSO) which can be quantified by checking the optical density via spectrophotometric analysis. A gradual increase in the optical density indicates that cells grow and proliferate on the scaffold material. This assay was done for all cryogels synthesized from two different crosslinkers. A comparison was made by establishing a control where cells were grown under 2-D conditions. All cryogels showed an increase in cellular metabolic activity for longer time duration when compared to 2-D control. Carrageenan-gelatin cryogels synthesized with glutaraldehyde as a crosslinker showed a gradual increase till 5th day of the analysis after which a drop was observed. The other type of carrageenan-gelatin cryogels synthesized with EDC-NHS, we observed the same trend but with decreased cell proliferation as compared to CGG. In case of 2-D control cells showed an increase in proliferation till 3rd day, after which a drop was observed. So, in all the cases the trend remains the same but showed variation in values of relative viability which is represented here in terms of optical density (Figure 11).

## 4. Discussion

CGG and CGE cryogels were fabricated by the cryogelation technique at −12°C for 16 h. Two different types of cryogel matrices were synthesized by using a combination of natural polymers carrageenan and gelatin with two different types of crosslinkers, that is, glutaraldehyde and EDC-NHS. EDC belongs to the class of zero-length crosslinker that does not become a part of linkage the part of linkage but modifies amino acid side-groups to permit crosslinking reaction and NHS is used to improve the EDC crosslinking [14, 32]. Rationale for using gelatin for the fabrication of the cryogel matrices is that it possesses Arg-Gly-Asp-(RGD-) like sequences which enhance cell adhesion and proliferation [15]. So, incorporation of gelatin promotes the cell attachment which is important for any type of matrix to be utilized for tissue engineering applications. Rationale behind using other polymer carrageenan was its potential in biomedical and tissue engineering applications. It is haemocompatible and was also used in bone tissue engineering application [25]. For the synthesis of an ideal scaffold, different concentrations of natural polymers such as carrageenan:gelatin were tried with crosslinkers, that is, glutaraldehyde and EDC-NHS. Final gels were fabricated using optimized concentrations of polymers and crosslinkers. It was observed that concentration more or less than the optimum concentrations leads to the formation of brittle, less elasticity, and soft or weak gels. The optimized carrageenan-gelatin cryogels obtained were elastic, porous, mechanically strong in nature and they did not get deformed when external pressure was applied or during the squeezing out of all solvent present inside the swollen cryogel. Optimized concentration of carrageenan in CGG was more than CGE which increases its stiffness and gelatin concentration was the same in both cryogels which was responsible for spongy and elastic nature. There is a possibility of different types of crosslinking reactions which can happen at the time of fabrication or synthesis of gels. Probable types of chemical reactions are shown by a schematic representation (Figure 1). In case of carrageenan-gelatin cryogel with glutaraldehyde as a crosslinker, amino groups present in gelatin may crosslink with aldehyde group of glutaraldehyde leading to the formation of imine. As gelatin is having both carboxylate and amino groups, there is a possibility of self-crosslinking which may lead to the formation of amide linkage. Carrageenan self-gelates at room temperature or may form interpenetrating network (IPN). Other possibility can be the formation of hydrogen bonding with glutaraldehyde. In case of carageenan-gelatin cryogel with EDC-NHS as crosslinker, self-gelation of carrageenan might take place or interpenetration during fabrication. In other case carboxylate group present in gelatin may get crosslinked with EDC resulting in the formation of an unstable o-acyl urea which again reacts with NHS and in the presence of amino group of the same gelatin chain or new gelatin polymeric chain leads to the formation of stable amide linkage [14, 33]. These possible bond formations were confirmed by FTIR which showed that peaks at different wave number correspond to particular linkage and functional groups. On physical examination both types of cryogels appeared stiff and mechanically stable indicating that they can be potential matrices for tissue engineering applications. Taking a closer look on the architecture on these matrices SEM analysis of both lateral and horizontal cross sections revealed that surface of these gel matrices was smooth and porous. 

Scaffolds exhibited large and interconnected pores which can allow the nutrient flow and waste exchange. Pore size of these matrices was in the range of 60–100 *μ*m which allows the efficient cell migration and accumulation of ECM. Large and interconnected pores also allow the flow of solvent or media which reduces the risk of cell death during high seeding density. Both types of cryogel matrices showed a good flow rate which is an indication of interconnected porous network. Good interconnected porous network allows an efficient nutrient transfer and gaseous exchange. These properties enhance the cell proliferation and cell survival on these matrices. During the growth of cells on the matrix they synthesize a large amount of ECM which can clog the pores leading to the death of cells. An efficient and interconnected and porous network of cryogels avoids cell death due to inefficient nutrient circulation and lack of oxygen. Furthermore both types of cryogel matrices had good pore volume indicating that cells can efficiently attach to the pore walls and proliferation with the production of large amount of ECM. High porosity of these matrices allows the efficient gaseous exchange which stimulates the cell growth even to the deeper areas of the gel. So, it can be inferred after estimating the flow rate, pore volume and porosity of these cryogels, that have good interconnectivity between pores which was observed more in CGE as compared to CGG. All these properties ratify the use of these cryogel matrices for tissue engineering applications. Density is dependent on mass and volume; thus it was observed that increasing the concentration of polymer also increased the density of the cryogel samples [12]. In case of CGG cryogels density was found little more than that of CGE. The density of these cryogels was found lesser than the density of water, which indicates that these matrices have porous architecture and they will not get immersed in water. Density measurements confirmed the presence of porous architecture which allows an efficient cell migration and nutrient flow.

After cyclic swelling and deswelling studies for both cryogels CGG and CGE the kinetics was obtained from the graph which showed a constant swelling behavior. There was not much difference between the kinetic of two different types of gels which means that they have reached their equilibrium approximately at the same time. But there were some differences in the swelling ratio which was found lesser in CGG than CGE. It indicated that CGE cryogel swells faster than in CGG. From the swelling and deswelling results it can be inferred that these gels do not show much changes when swelled and deswelled repeatedly. These results indicate that they can be potential matrices for tissue engineering applications where scaffolds should not show altered behavior when swelled with media or deswelled during cell culture experiments. X-ray diffractogram of these gel matrices showed some peaks in blend of carrageenan and gelatin which were similar to the peaks observed in the neat carrageenan which confirms its crystallinity. Neat gelatin did not show any peaks which is an indication of its amorphous nature. In blend both polymers are present which implied that when the concentration of carrageenan increased in case of CGG, intensity of the crystal peaks was more than that in CGE but not when compared to neat carrageenan polymer. When blending occurs between crystalline carrageenan polymer with the amorphous gelatin polymer, these polymers may get interpenetrated and there could be a possibility of the reduction of crystalline peaks intensity. Thus it can be inferred after blending that carrageenan did not show more number of sharp peaks. TGA thermogram of weight loss and temperature revealed that initially at first 5% and 10% weight loss occurred due to the loss of moisture from the cryogel sample having two different crosslinkers. Second curve was observed in the range of 250–400°C and associated with the breakage of bonds formed during crosslinking or degradation of functional groups involved in bond formation. Any further weight loss in TGA was not observed beyond 750°C. Thus it can be assumed that decomposition of carbonaceous content of the precursor occurred up to 750°C and final residue yield also remained in the end. Therefore, it can be inferred that these matrices can even have applicability at high temperatures which indicates their utility for other applications also. Compression analysis was done for the determination of mechanical strength of cryogels (CGG, CGE).

In tissue engineering, maintenance of mechanical integrity is a significant factor during fabrication of scaffold [15]. These cryogels (CGG and CGE) could be compressed up to 90% of their original length without showing any permanent deformation or development of cracks. These cryogels have gelatin as one of the polymers which provides elastic behavior and when blended with carrageenan it gives strength to the cryogels. Thus, these synthesized cryogels are spongy and regained their original shape after immersing them again in solvent. Tissue engineering is a regenerative strategy which focuses on the use of 3D scaffolds as tissue replacements. So, after the scaffold is implanted, it should ideally degrade at a pace which should match the regeneration of the native tissue. Rate of degradation can be modulated by using specific biodegradable polymers with optimized crosslinker concentration. During the fabrication of the scaffold, its degradation parameters were studied and optimized according to the usage for tissue engineering applications. CGG cryogel synthesized by using glutaraldehyde as crosslinker showed more degradation rate as compared to CGE where EDC-NHS was used as a crosslinker. Weight loss of the gels occurring due to *in vitro* degradation indicates the collapse of internal structure architecture. According to the previous studies, gelatin degrades quickly but if it gets crosslinked with other polymer, then the crosslinking decreases the rate of degradation [15]. In this research work, we have crosslinked gelatin with carrageenan which leads to decrease in the rate of degradation. Overall the rate of degradation was slow but this rate can further be enhanced due to the presence of different enzymes and factors under *in vivo* conditions. As all the scaffolds fabricated for tissue engineering applications are intended for *in vivo* usage, degradation rate of synthesized scaffolds seems to be an optimal.

Rheology study of the carrageenan-gelatin cryogels inferred that scaffolds bear stress without any permanent deformation and were mechanically stable with viscoelastic nature at constant temperature (37°C) and at constant strain (0.1%) for both dry and wet states. Rheological data includes three major parameters such as storage modulus, loss modulus and phase angle. Storage modulus of CGG and CGE in both dry and wet state remains constant throughout the run, indicating that initial elasticity of the material increased with the applied force and then it showed resistance to the same force, which means it does not alter the elastic component of the material. Storage modulus of both the cryogels in dry state was observed more as compared to the wet state which showed that elasticity of the material decreases in wet state (Figures 9(a), 9(b), 9(c), and 9(d)). Loss modulus represents viscous behavior of the material. Here, loss modulus was observed lesser than the storage modulus for both the cryogels in dry and wet conditions which indicates that material is less viscous in nature. Loss modulus in dry condition for both cryogels was found to be more as compared to the wet condition and more variations or fluctuations were seen in wet state. Thus, in wet state viscosity of the materials was less. Phase angle is the difference between stress and strain in an oscillatory test. Phase angle in dry state for both the cryogels was decreasing and showed more variation in wet state indicating that gels are more elastic but have less viscous behavior (Figures 9(a), 9(b), 9(c), and 9(d)). On further analysis storage and loss moduli become constant and with no change in phase angle which showed the equilibrium state of cryogels. Thus, these cryogel matrices showed good viscoelastic properties which can be used for various tissue engineering applications. Among the prerequisites of a tissue-engineered scaffolds its biocompatibility under *in vitro* conditions is important [12]. Biocompatibility of the scaffold is represented by proliferation and production of ECM on them. After seeding Cos-7 cells on scaffolds CGG and CGE cells get adhered and start proliferating which was confirmed by SEM images. After 2nd day it was observed that cells adhered and morphology of cells becomes flattened. Few cells were observed in their dividing state along with the secretion of ECM. After 7th day it was found that cells started growing in clusters all over the matrices and ECM production was more in comparison to that of the 2nd day. Cellular adherence to the scaffold matrices was also checked by fluorescence microscopy. Seeded scaffolds were stained by PI, which penetrates the cell membrane and stains nucleus. Nuclei of the cell can be observed at an excitation wavelength of 536 nm and an emission of 617 nm. Staining results confirmed that the cells adhered properly to the cryogel matrix. It was observed and confirmed by PI nuclear stain. Cells were uniformly distributed and proliferated on 5th day of cell culture on the surface of cryogel sections.

Cellular proliferation and ECM production are an indication that these matrices are biocompatible and support the synthesis of ECM. Results also signify that glutaraldehyde used as a crosslinker at low concentration did not affect the growth of cells. So synthesized cryogel matrices show the potential for tissue engineering applications. Metabolic activity of the cells on the scaffold was confirmed by MTT assay. In case of control, that is, two-dimensional (2-D) system the metabolic activity of fibroblast cells (Cos-7) increased till 3rd day and then it started declining and was observed very less after 10th day of cell culture experiment which is related to the increase and decrease in an optical density indicating cell adhesion and proliferation. Decrease in metabolic activity was observed in 2-D as compared to the synthesized 3D scaffolds. On a two-dimensional (2-D) system, cells grow and proliferate to occupy the available surface area and reached to the confluency within 5 days. After cells occupy the available surface, they tend to die due to contact inhibition, accumulation of toxins, and lack of nutrients which leads to the peeling off of the monolayer. But in case of three-dimensional (3D) system, surface area available for cell growth and proliferation is more which results in extended proliferative activity on these matrices for longer duration of time. On day zero (after 6 h of seeding) Cos-7 cells adhering on 2-D and CGG matrix showed similar metabolic activity but in case of CGE matrix cells might be less adhered and take more time to proliferate and therefore less metabolic activity was observed as compared to 2-D. Results indicate that scaffolds support proliferation of cells which are considered to be biocompatible.

## 5. Conclusion

In conclusion in this work we have fabricated polymeric cryogel matrix from two natural polymers having two different crosslinkers by cryogelation technique. Matrices found to be supermacroporous and having interconnected porous architecture with good mechanical strength and viscoelastic behaviour. These cryogels also showed biodegradable nature, constant swelling kinetics, and swelling behaviour. Cell matrix interaction was also done which inferred good cell adhesion, proliferation, and secretion of ECM on matrices. Thus, these carrageenan-gelatin matrices with two different crosslinkers such as glutaraldehyde and EDC-NHS showed promising results which fulfilled the criteria of a potential scaffold for tissue engineering applications.

## Figures and Tables

**Table 1:** Characteristics of carrageenan-gelatin cryogels (CGG and CGE) prepared by using different crosslinkers.

Cryogels	Average % of porosity	Pore volume	Average swelling ratio	Flow rate (mL/min)	Density (g/cm^3^)		Elastic modulus (kPa)		TGA			Average degree of degradation (%)
					Dry	Wet	Dry	Wet	5% weight loss	10% weight loss	Final residue (%)	
CGG	77.12 ± 1.73	12.78 ± 0.96	3.70 ± 0.10	3	0.00266	0.0360	10.98	0.068	70.31°C	228.27°C	1.54%	50.55 ± 5.49
CGE	83.73 ± 3.99	14.3 ± 0.52	10.25 ± 0.74	4	0.00207	0.0422	4.8	0.038	70.31°C	177.73°C	1.23%	34.17 ± 2.52
